# On-Site Deployment of an Air-Liquid-Interphase Device to Assess Health Hazard Potency of Airborne Workplace Contaminants: The Case of 3-D Printers

**DOI:** 10.3389/ftox.2022.818942

**Published:** 2022-03-25

**Authors:** Boowook Kim, Jae Hoon Shin, Hoi Pin Kim, Mi Seong Jo, Hee Sang Kim, Jong Sung Lee, Hong Ku Lee, Hyuk Cheol Kwon, Sung Gu Han, Noeul Kang, Mary Gulumian, Dhimiter Bello, Il Je Yu

**Affiliations:** ^1^ Institute of Health and Environment, Seoul National University, Seoul, Korea; ^2^ Institute of Occupation and Environment, KCOMWEL, Incheon, Korea; ^3^ Aerosol Toxicology Research Center, HCTm, Icheon, Korea; ^4^ Toxicology Laboratory, Sanghuh College of Life Science, Konkuk University, Seoul, Korea; ^5^ Division of Allergy, Department of Medicine, Samsung Medical Center, Sungkyunkwan University School of Medicine, Seoul, South Korea; ^6^ Haematology and Molecular Medicine, University of the Witwatersrand, Johannesburg, South Africa; ^7^ Water Research Group, Unit for Environmental Sciences and Management, North West University, Potchefstroom, South Africa; ^8^ Department of Biomedical and Nutritional Sciences, University of Massachusetts, Lowell, MA, United States; ^9^ HCT. Co., Icheon, Korea

**Keywords:** air-liquid-interphase, workplace, 3-D printer emission, silver nanoparticles, biomonitoring, TNF-α mRNA, inflammasome

## Abstract

Biomonitoring of workers is an approach of evaluating workers’ exposure to chemicals and particulate matter by measuring biomarkers of parent chemicals, their metabolites, and reaction products in workers’ biospecimens. Prerequisites for biological monitoring in the workplace include permission to enter the workplace, approval of the study plan from the IRB (Institutional Review Board), and obtaining consent from workers. Because of the complex legal process involved in biomonitoring, few studies have been conducted so far on biomonitoring of workers’ exposures to nanoparticles and other hazards from emerging materials and advanced nanotechnologies. We have developed a cell-based biomonitoring device that can evaluate acute cytotoxicity and various other effect biomakers, such as inflammation, at realistic workplace exposure. This device is based on air–liquid interphase (ALI) and can be used to evaluate cell toxicity and early effect biomarkers along adverse outcome pathways. Following exposure of A549 lung epithelial cells in ALI to workplace air for 1–2 h, the cells were processed to assess the induction of inflammatory and cell damage biomarkers. Initially, we estimated the deposition rate of nanoparticles in the transwell by exposing the cell-free ALI device to silver nanoparticle aerosols (AgNP 20–30 nm) for 2 h in the laboratory. Then A549 lung epithelial cells cultured on the transwell in the ALI device were exposed to AgNP nanoaerosols for 2 h and evaluated for cytotoxicity and induction of mRNAs of pro-inflammatory cytokines IL-1b, IL-6, and TNF-α. Then the cells in the ALI device were exposed to 3-D printer emissions at the workplace and evaluated for the same matched endpoints. The mRNA levels for IL-1b, IL-6, and TNF-α increased significantly at the end of 2-h exposure of A549 cells to the positive control AgNP aerosols. These mRNAs, as well as LDH and microprotein concentrations, increased even more after 24-h post-exposure incubation (*p* < 0.05). Cytotoxicity evaluation of 3-D printer emissions at 810 and 957 μg/m^3^, which was more than 80 times higher than the airborne total suspended particulate concentrations in the workplace air (9–12.5 μg/m3), suggested no significant acute cytotoxicity at the end of 2-h exposure to 3-D-printing emission, as well as at 24-h post-exposure incubation. Hyperspectral microscopic observation showed that 3-D printers emitted particles to be attached to A549 cells after 2-h exposure, and many particles were internalized by A549 cells after 24 h of post-exposure incubation. The mRNA expression of pro-inflammatory cytokine IL-1b and IL-6 increased significantly after 2-h exposure to 3-D printer emissions and after 24-h incubation (only IL-6). In contrast, the expression of TNF-α mRNA decreased significantly after 2 h of exposure to 3-D printers and decreased even more after 24-h post-exposure incubation. These results support the use of cell-based ALI devices for direct assessment of airborne hazards in the workplace, for probing toxicological properties of airborne contaminants using adverse molecular pathways, and for guiding study design for workplace biomonitoring. ALI devices can bridge conventional exposure assessment with cellular toxicity testing platforms for hazard and risk assessment.

## Introduction

Biomonitoring is an important approach to evaluate workers’ exposures to chemicals and particulate matter in the workplace by measuring biomarkers, including parent chemicals, their metabolites, and reaction products in workers’ biological specimens. Prerequisites for biological monitoring in the workplace include permission to enter the workplace, approval of the study plan from the IRB (Institutional Review Board), and obtaining consent from workers. Because of the complex legally required process for biomonitoring studies, few studies have been conducted so far for biomonitoring of workers’ exposures to engineered nanoparticles, advanced materials, and additive nanomanufacturing. Similar principles to biomonitoring are applied when conducting health surveillance studies, although the local legal context may vary. We conducted several biomonitoring studies of workers in nanomaterial manufacturing and handling workplaces, along with exposure assessment. A silver nanoparticle manufacturing workplace was investigated in an early study for exposures to Ag (NP) and related health effects in workers. Personal sampling for assessing Ag (NP) exposures, as well as blood and urine specimens, were collected from the workers to measure the internal silver concentration (Lee et al., 2011). A multiwalled carbon nanotube (MWCNT) manufacturing workplace was studied for workers’ exposure to MWCNT and related health effects. Exposures to CNTs were assessed *via* personal and area exposure measurements; lung function testing was conducted to evaluate lung function; and blood and exhaled breath condensates (EBCs) were collected from the exposed workers to estimate any changes for relevant biomarkers (Lee et al., 2014). These kinds of biomonitoring studies are always expensive and complicated by the need to secure prior IRB approval, workplace access, workers’ consent, and the involvement of medical doctors. In the case of carbon nanomaterials, including MWCNT, measuring their internal concentration in blood was (and still is) a daunting task, although measuring metal nanoparticles such as silver was possible. Other general health effect markers such as clinical hematology and blood biochemical markers are often of limited use, especially for emerging hazards with no past exposure history (short exposure latency) and for low exposures, as well because they are not sufficiently sensitive. Furthermore, many toxicological endpoints from animal testing results are difficult to confirm in human working populations. Many toxicological endpoints in animal studies (e.g., histology or tissue-specific analysis at the organ level) are extremely invasive and not possible to apply in humans, except under extreme circumstances. Noninvasive biomarkers identified from animal studies or *in vitro* studies are seldom validated (or found) in human biomonitoring studies. Besides interspecies variations in the biological response, a major difficulty is that humans have a much wider range of phenotype variation, lifestyle factors, and comorbidities that require much larger samples to be able to detect health effects of a comparable magnitude with animal experiments. Moreover, animal studies tend to use much higher doses than what is typical of workers’ exposures in workplaces.

More recent developments in alternative testing strategies encourage the exploration of mechanistic injury pathways *in vitro* in cells, as well as improved approaches and strategies to mimic *in vivo* studies with minimal reliance on the use of animals. One such improvement on alternative inhalation testing is the use of the air–liquid interphase (ALI) cell-based approach. The delivery of airborne nanoaerosols to the cells in the ALI mimics human inhalation exposure to such nanoparticles with regard to important considerations such as deposition mechanisms, dose rate/particle flux, particle aggregation/agglomeration, and cellular microenvironment. In the ALI system, lung cells, alone or in co-cultures with other lung-associated cells, are exposed to aerosolized particles similar to inhalation. This delivery mode avoids the well-known artifacts associated with the preparation of powdered nanoparticle dispersions, including particle aggregation, agglomeration, and corona formation, and produces a dosimetrically more realistic exposure situation. In addition, gaseous pollutants, often co-present with airborne nanoaerosols, may be lost when particles are collected on filters and then dispersed and resuspended in cell culture media. Therefore, it can be argued that the delivery of airborne pollutants (aerosol and/or vapors) to cells through the ALI exposure system is more physiologically representative of human inhalation exposures than the conventional *in vitro* methods (of submerged cells). The use of ALI systems may also reduce the number of animals used in inhalation studies and may, at times, provide more relevant information for *in vivo* toxicological mechanisms in humans, some of which might differ or do not exist in animal models due to variations in their physiology (Tralau et al., 2015). In addition, the hybrid features of the ALI system—combining relevant airborne exposures from an occupational/environmental setting with a physiologically relevant exposure delivery to representative lung cells—may provide highly relevant and cost-effective data for risk assessment purposes.

In this report, we employed the ALI system containing A549 lung epithelial cells to probe the respiratory hazards of airborne 3-D printer emissions directly at the workplace by assessing cell viability, cell barrier integrity, cellular energetics, cell necrosis, and the production of important pro-inflammatory markers in the inflammasome/NF-kB pathway (IL-6, IL-1b, and TNF-α) immediately at 2-h workplace exposure and after 24-h post-exposure cell incubation. The findings suggest that the ALI system can provide insightful input into the likely adverse health effects of workplace exposures to workers and can guide targeted human biomonitoring efforts, as well as for risk assessment.

## Materials and Methods

### Cell Culture

A549 cells, obtained from ATCC (American Type Culture Collection, #CCL-185, Manassas, VA, United States), were cultured in an RPMI 1640 medium and supplemented with 10% fetal bovine serum (FBS) and 100 units of penicillin and streptomycin at 37°C and 5% CO_2_ in an incubator. The cells were detached by 0.25% trypsin EDTA and counted and re-plated on the transwell (Cell culture insert, transparent PET membrane, 4.7 cm^2^, 0.4 pore size, Falcon Cat No353090) at a density of 1.5 × 10^5^ cells/ml. The volumes of the medium at the apical and basal sides were 1 and 2 ml, respectively (Kim et al., 2013). Cells became confluent after one day; then, they were cultured overnight after removing the medium from the apical side of the transwell.

### *In Vitro* ALI Exposure System

**FIGURE 1:**
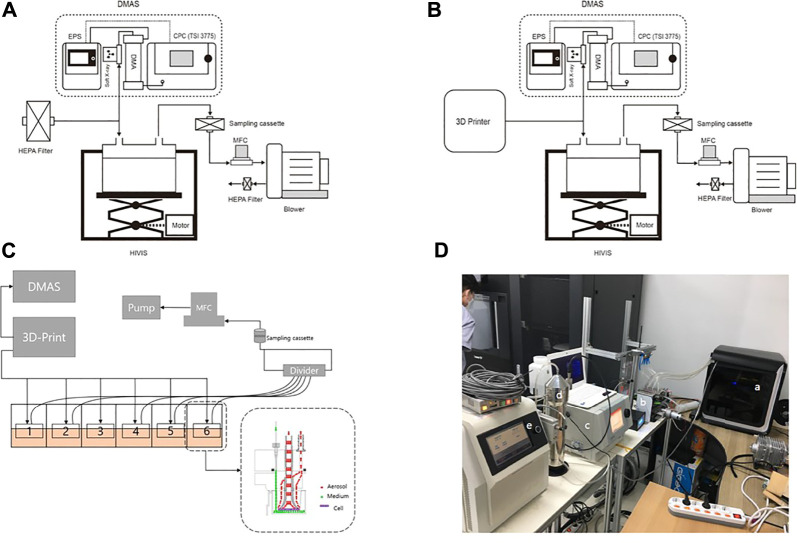
HIVIS system for 3-D printer emission exposure. DMAS, differential mobility analyzing system; EPS, electrical particle sensor; HEPA filter, high-efficiency particulate air filter; MFC, mass flow controller. **(A)** Control; **(B)** 3-D printer emission exposure; **(C)** scheme of 3-D printer emission exposure to 6-transwell plate; **(D)** experimental setup. (a) 3-D printer; (b) HIVIS; (c) CPC; (d) DMA; (e) EPS.

The HIVIS ALI exposure system (HCTm *in vitro* inhalation system, HCTm, Co. Icheon, Korea) consisted of an aerosol exposure unit accommodating commercially available (6-well) transwell culture plates, aerosol monitoring systems (differential mobility analyzer, DMAS; electrical particle sensor, EPS; and other sensors) and air sampling unit, and a mass flow controller (MFC) (Figures 1A-D). Individual trumpets for each transwell insert delivered airborne nanoaerosol emissions individually to each insert, not the plate as a whole (Figure 1C). For the 3-D printer emission exposure study, emissions were delivered in a dynamic mode to the manifold of the HIVIS exposure unit by placing a 6-well transwell plate directly after removing the cover. The 3-D emissions were delivered to each transwell at a flow rate of 10 ml/min for 2 h through a mass flow controller (MFC, 60 ml/min total flow). The 10 ml/min flow rate does not affect cell viability, as determined in separate experiments (Supplementary Table S2). The exhaust aerosols were captured on the sampling filter, which is then used to estimate aerosol air concentration located in front of the MFC.

### Deposition Rate Estimation to the Transwell

The deposition rate to the HIVIS was estimated by using silver nanoparticle aerosols (AgNPs, 20–30 nm). AgNPs (20–30 nm) were generated from an aerosol generator consisting of a small ceramic heater connected to an AC power supply that was housed within a quartz tube furnace (Ji et al., 2007; Sung et al., 2009; Kim et al., 2021). The heater dimensions were 50 × 5 × 1.5 mm, and a surface temperature of about 1,500°C within a local heating area of 5 × 10 mm^2^ was achieved within about 10 s. The source material, silver wire (100 mg, 99.99% purity, 0.5-mm diameter, Higgslab Co., Ltd., Korea), was placed on a ceramic heater at the highest temperature point. The quartz tube was 70 mm in diameter and 140 mm in length. Clean (dry and filtered) air was used as the carrier gas, and the gas flow was maintained at 3.5 L/min (Re = 572, laminar flow regime) using an MFC (AERA, FC-7810CD-4 V, Japan) to deliver AgNP aerosols to a mixing chamber. AgNP aerosols taken from the mixing chamber were exposed to the MCE filters (size: 37 mm and pore size 0.45 μm, SKC, United Kingdom) placed in the transwell with a flow rate of 60 ml/min. The flow rate to each transwell was 10 ml/min. MCE filters in the transwells were stationed with a stainless cassette (thickness and diameter) not to fold or shrink or not to penetrate the backside of the filter surface. After the designated exposure time, filters were digested on a hot plate (PerkinElmer, Concord, ON, Canada) using nitric acid (Fluka, Lot; BCBM5181V). The mass concentrations of AgNP were determined chemically by using an atomic absorption spectrophotometer (AAS, Perkin-Elmer 900 T, Waltham, MA, United States). In addition, a downstream filter in the sampling cassette was also analyzed for air AgNP mass concentration in the transwell.

### Dose Estimation to the Cells in the Transwell

The deposited concentration was calculated by the equation below:

$$100\%\;D\left(\frac{mg}{transwell}\right)=M\;\left(\frac{mg}{m^{3}}\right)\times F\left(L/min\right)\times T\left(min\right)\times 10^{-6}\left(\frac{m^{3}}{mL}\right)$$ (1)

where 100% D is the total deposited nanoparticle dose on transwells (mg/transwell); M is the average aerosol concentration (mg/m^3^); F is the flow rate (L/min); and E is the exposure duration (min) 10^–6^, correction factor between m^3^ and ml.

The deposited dose D’ was calculated based on Eq. 2

$$D^{\prime }\left(\frac{mg}{traswell}\right)=100\%D\left(\frac{mg}{transwell}\right)\times R$$ (2)

where D’ is the deposited dose 100% D, calculated from Eq. 1. R is the system deposition rate for nanomaterials with similar size (30% for 30 nm silver particles in HIVIS system).

The calculations assume that the density contribution is minimal at the diffusion dominant deposition (Table 1).

**TABLE 1:** Primer sequences used for RT-PCR in this study. Abbreviations: IL-1β, interleukin-1β; IL-6, interleukin-6; TNF-α, tumor necrosis factor-α; GAPDH, glyceraldehyde 3-phosphate dehydrogenase.

Gene	Primer sequence 5′–3′
IL-1β (Human)	(F) TAC CTG AGC TCG CCA GTG AAA T
	(R) CCT GGA AGG AGC ACT TCA TCT GTT
IL-6 (Human)	(F) ACA GCC ACT CAC CTC TTC AGA AC
	(R) TTT TCT GCC AGT GCC TCT TTG C
TNF-α (Human)	(F) AAG CCC TGG TAT GAG CCC ATC TAT
	(R) AGG GCA ATG ATC CCA AAG TAG ACC
GAPDH (Human)	(F) GAC CCC TTC ATT GAC CTC AAC TAC
	(R)ATG ACA AGC TTC CCG TTC TCA G

### Measurement of Cytotoxicity

A549 cells cultured with an RMPI-1640 medium were washed three times with phosphate-buffered saline (PBS) and incubated with RPMI 1640 without serum in the ALI state (i.e., 2 ml of RPMI in the basal side and no medium on the apical side). The cells were exposed to the 3-D printer emissions for 2 h at an airflow rate of 10 ml/min, and particle numbers were counted on the inside of the 3-D printer by DMAS. Silver nanoparticles (AgNP, 20–30 nm) generated from a hot-plate generator were used as a positive control (Mendes et al., 2017; Stefaniak et al., 2017; Vance et al., 2017). Two ALI exposure experiments (yielding two sets of 6 wells) were conducted, each 2 h long. During these experiments, we simultaneously monitored the particle number and size distribution by DMAS, as well as the mass concentration of 3-D printer emissions delivered to the cells on the transwells. During the exposure period, CO_2_ was not supplied to the cells, and the control cells were similarly exposed to HEPA-filtered air for 2 h. After 2-h exposure, the culture medium RPMI 1640 on the basal side was collected and stored for further analysis. The cells from two transwells were detached by 0.25% trypsin EDTA and evaluated for cytotoxicity by trypan blue exclusion by counting 200 cells. The cells on the other four transwells were supplied with RPMI 1640 with 10% serum and incubated for 24 h at 37°C and 5% CO_2_ in an incubator. After this post-exposure incubation, the medium in the basal side was collected into 15 conical tubes and stored in the −80°C refrigerator for further analysis. Cytotoxicity endpoints included cell necrosis by the lactate dehydrogenase (LDH) assay, total protein, and microprotein using a biochemistry analyzer (7,080 Automatic Analyzer, Hitachi High-Technologies Co., Tokyo, Japan).

### Determination of mRNA Expression Level for Inflammatory Cytokines Using a Real-Time Polymerase Chain Reaction

A549 cells were collected with a cell scraper after exposure to AgNP or ABS 3-D printer emissions for 2 and 24 h, and total RNA was extracted from A549 cells using a TRIzol reagent (Ambion, Austin, TX, United States) to evaluate mRNA expression levels of inflammatory cytokines (*n* = 3 wells per group). The TOPscript RT DryMIX kit (Enzynomics, Daejeon, Korea) was used for the reverse transcription of RNA (2 µg). The final volume of 20 µl was incubated at 37°C for 5 min, 50°C for 60, and 95°C for 5 min. A total 20 µl volume of PCR solution, including 10 µl of 2 × Real-Time PCR Smart mix (Solgent, Daejeon, Korea), 8 µl of RNase-free water, 1 µl of cDNA sample, and 0.5 µl each of 10 µM forward and reverse primers (Bionics, Seoul, Korea), was analyzed by real-time polymerase chain reaction (RT-PCR) system (Roche LightCycler^®^ 96 System, Basel, Switzerland) to evaluate the mRNA gene expression level. The thermal conditions of PCR cycles were as follows: 95°C for 15 min, followed by 60 cycles of denaturation at 95°C for 10 s, annealing at 60°C for 10 s, and extension at 72°C for 10 s. The mRNA expression level was quantified using the 2-ΔΔCt method and normalized using the GAPDH mRNA level. The primer sequences were designed using the Amplifx software, and sequence details are shown in Table 1.

### Workplace 3-D Printer Exposure

The workplace manufactured computer-aided design (CAD) objects by 3-D printers consuming 46 and 102 g of ABS filament (containing 95–100% ABS; 0–5% stabilizer; melting point 180–200°C; Plasil, 3Dink Inc., Yangju, Korea) for printers 1 and 2, respectively, to manufacture headphone hangers (Supplementary Figure S1).

Supplementary Figure 2 illustrates the workplace layout, sampling sites, and other contextual information. Four male workers were located in front of Tables 1–4. The workers were computer programmers involved in developing the facility management system and UR robot operation. The UR robots were not operated on the day of the tests. The dimension of the room was 6.75 m × 11.25 m × 2.72 m (206.55 m^3^). The workplace had only one entrance door and no window and was equipped with three fresh air inlets and three returns located on the ceiling. One ceiling-mounted air conditioner unit was operated during the workday. The manufacturing CAD objects consumed 46 and 102 g of ABS filament (containing 95–100% ABS; 0–5% stabilizer; melting point 180–200°C; Plasil, 3Dink Inc., Yangju, Korea) for printers 1 and 2, respectively, to manufacture headphone hangers (Supplement Supplementary Figure S1).

**FIGURE 2:**
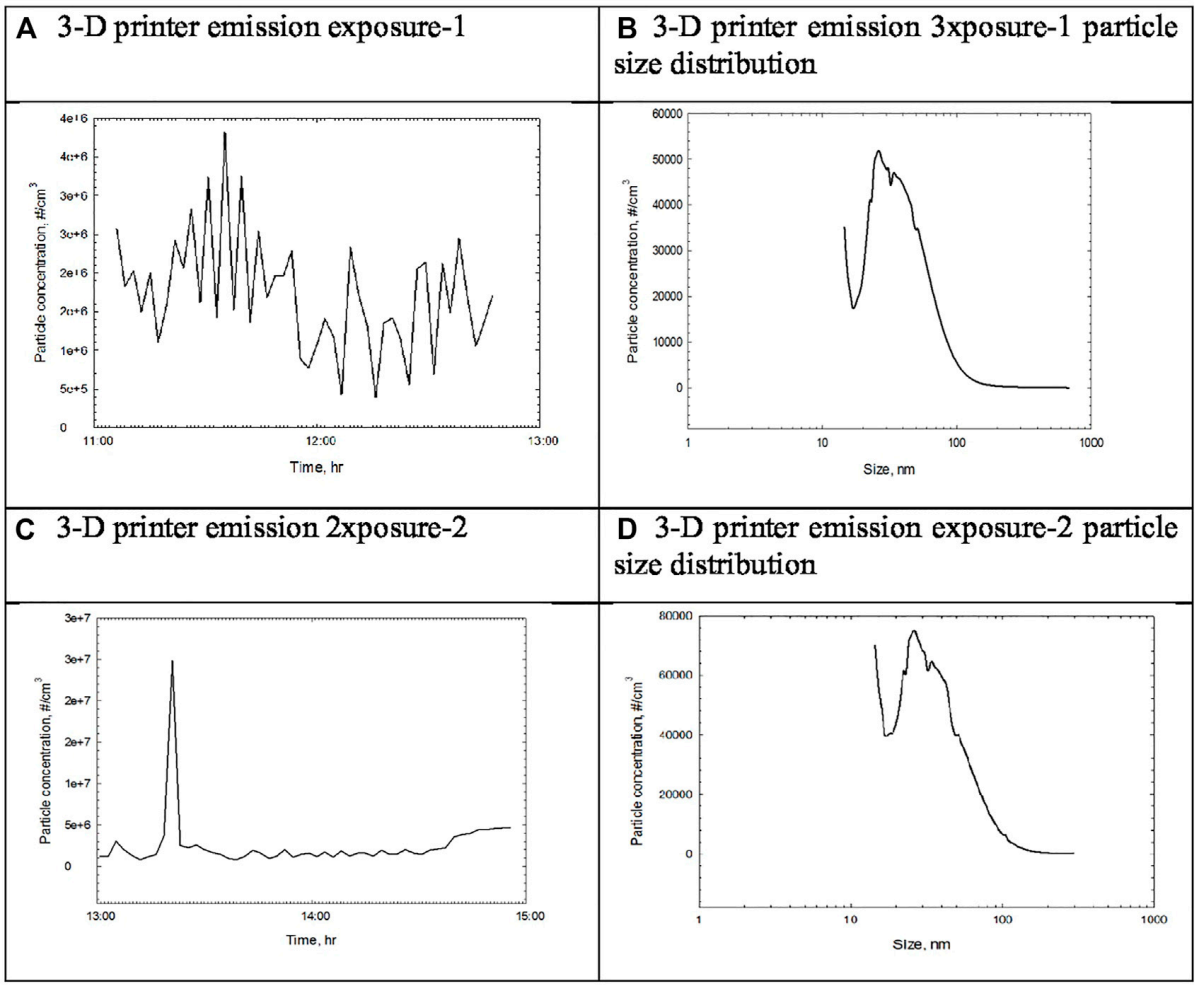
Particle concentration and size distribution measured by DMAS during the 2-h exposure of 3-D printer emission. **(A)** 3-D printer emission exposure-1. **(B)** 3-D printer emission eposure-1 particle size distribution. **(C)** 3-D printer emission exposure-2. **(D)** 3-D printer emission exposure-2 particle size distribution.

**TABLE 2:** Deposition rate of 20–30 nm AgNP nanoaerosols to the transwell.

Trans-well number	(A) Concentration of downstream filter (ng)	(B) Flow rate to 6-transwell (ml/min)	(C) Exposure duration to transwell (min)	(D) Air concentration (ng/m^3^) = A/(B x C)	(E) Concentration Ag in the filter placed in the transwell (ng)	(F) Flow rate to each transwell (ml/min)	(G) 100% deposition to the filter in the transwell (ng) = D x C x F/10^6^	(H) % of deposition = E/H x 100%
1	3,330.23	60	120	463,532	166.94	10	555	30.1
2					163.01			29.4
3					162.53			29.3
4					143.70			25.9
5					152.76			27.5
6					156.59			28.2
Average ± SD					157.59 ± 8.5			28.4 ± 1.54

**TABLE 3:** AgNP aerosol concentration during 2-h exposure.

	AgNP exposure 1	AgNP exposure 2
Total concentration (#/cm^3^)	1.73 × 10^6^ ± 7.40 × 10^5^	2.50 × 10^6^ ± 3.35 × 10^6^
GM (nm)	35.18	36.58
GSD	1.64	1.68

(mean ± S. D), GM, geometric mean; geometric standard deviation.

**TABLE 4:** Mass concentration of AgNP aerosol sampled during 2-h exposure.

Group	Ag Concentration in filter (ng)^#^	Flow rate (cc/min)	Sampling time (min)	Mass concentration (μg/m^3^)
2-h exposure	703.40	60	120	98
24-h post-exposure	812.87	60	120	113

#Measured by atomic absorption spectrophotometer.

### Hyperspectral Imaging of ABS Particles

**FIGURE 3:**
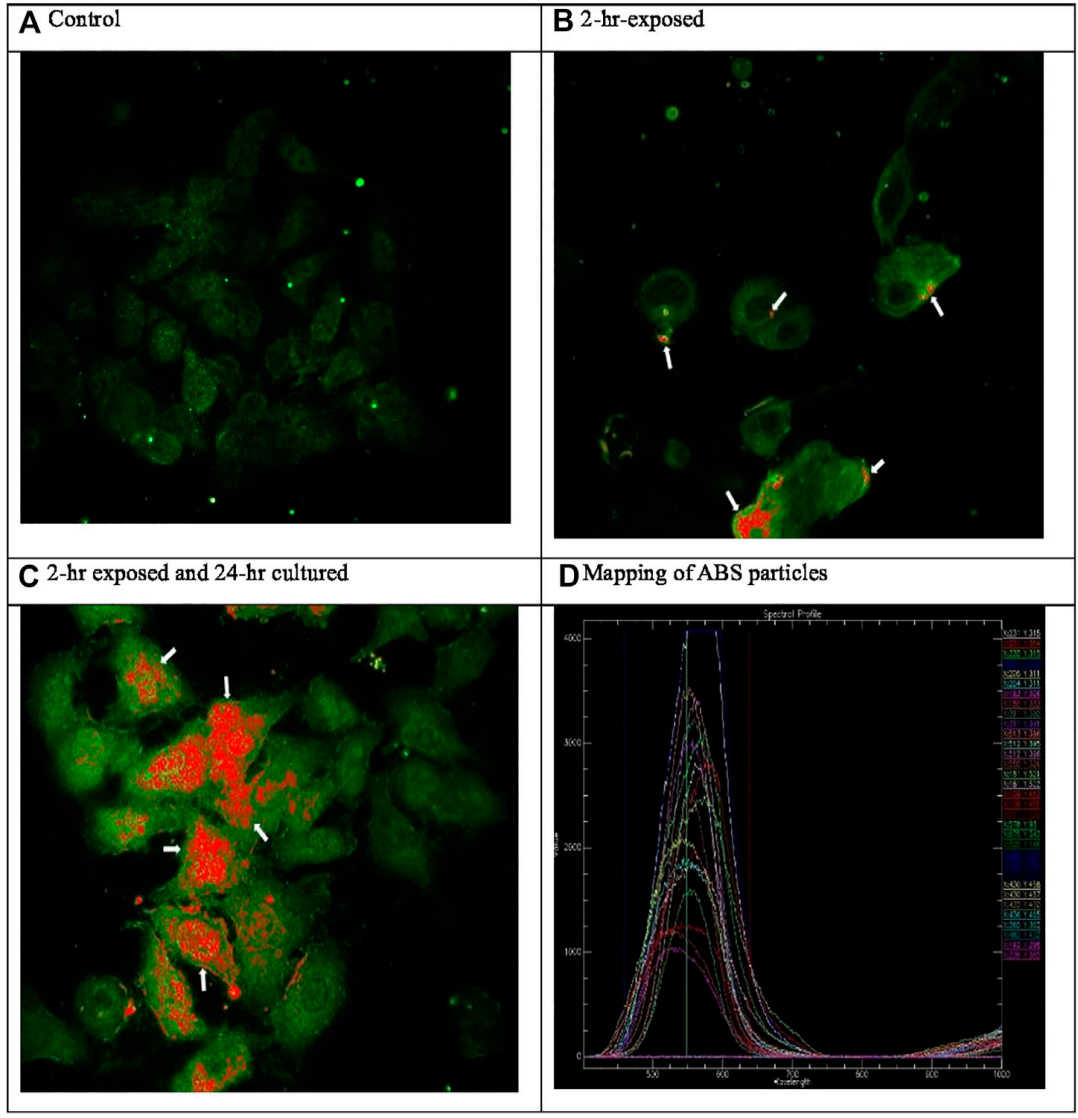
Hyperspectral microscopic images of ABS particle exposed A549 cells (400x). ABS particles deposited to the glass coverslips were scanned and obtained hyperspectral profiles (D). **(A)** Control. **(B)** 2-h exposed. **(C)** 2-h exposed and 24-h cultured. **(D)** Mapping of ABS particles.

A glass coverslip without culturing A549 cells was placed in the transwell to deposit ABS particles on the glass coverslip for 2 h. The ABS particle deposited coverslip was scanned to reference the hyperspectral profile (Figure 3A). A glass coverslip was placed in the transwell, and A549 cells were cultured on the glass coverslip, as well as the transwell. A549 cells on the coverslips in the transwell were exposed to 3-D printer emissions for 2 h (Figure 3B). For the post-exposure, A549 cells on the coverslips were cultured for 24 h after 2-h exposure to 3-D printer emission (Figure 3C). The A549 cells on the coverslips were fixed with 4% formaldehyde in PBS and examined under an optical system consisting of high signal-to-noise, dark field-based illumination optics adapted to an Olympus BX-41 microscope (CytoViva, Auburn, AL). The hyperspectral profiles for ABS particles (Figure 3D) were used to obtain ABS particle images in the A549 cells. Hyperspectral imaging can provide a qualitative spectral analysis of nanosized materials imaged using a dark-field-based microscope system and can also analyze biological nanoscale samples. The characteristics of the ABS particles, which scatter a significantly greater amount of light than the surrounding cell, were also assessed using the enhanced-darkfield optical system (Kim et al., 2012; Han et al., 2015; Kim et al., 2016).

## Results

### Deposition Rate Estimation

A representative result for the deposition rate obtained from AgNP aerosol deposition to the filters in the transwells is presented in Table 2. The result showed 28.4 ± 1.54% deposition, similar to the MPPD (multiple pathway particle dosimetry) model 3.04 (Anjilvel and Asgharian, 1995), which shows 28–26% deposition for 20 and 30 nm AgNP.

### Cytotoxicity Study of 3-D Printer Emission

In this study, 3-D printer emissions and positive control AgNP aerosols were directly exposed to the A549 cells cultured in the HIVIS ALI system. During two times of 2-h exposure, 1.73 and 2.50 × 10^6^ particles/cc were emitted for exposure-1 and exposure-2, respectively (Table 3), and mass concentrations captured on the filter during 2-h exposure were 810 and 957 μg/m^3^ for exposure-1 and exposure-2, respectively (Table 4). The particle number and size distribution are shown in Figure 2. The positive control AgNP mass concentrations were 98 and 113 μg/m^3^ for 2-h exposure and thereafter 24-h incubation, respectively (Table 5). These 3-D emission concentrations are more than 80 times higher than the TSP concentrations (8–11 μg/m^3^) measured in the workplace air. A 2-h exposure and thereafter 24-h post-exposure cell incubation did not affect cell viability, LDH release, microprotein, and total protein release into the culture medium (Table 6). The exposure of 3-D emitted particles to A549 cells was confirmed visually by a hyperspectral microscope (Figure 3). Some ABS particles were bound or taken by A549 cells after 2-h exposure, and many ABS particles internalized into A459 cells after 24-h post-exposure.

**TABLE 5:** Mass concentration of 3-D printer emission sampled during 2-h exposure.

Group	Filter weight (Before)	Filter weight (After)	Flow rate (cc/min)	Sampling time (min)	Mass concentration (μg/m^3^)
Control	11.894	11.895	60	120	93
Exposure 1	12.705	12.714	60	151	957
Exposure 2	12.194	12.199	60	96	810

**TABLE 6:** Cytotoxicity of 3-D printer emission to A549 cells. Two independent sessions of HIVIS exposure were conducted.

A. Positive control AgNP.														
Positive control AgNP														
		2-h exposure with AgNP							24-h post-exposure with AgNP				
		**Control**			**AgNP**				**Control**		**AgNP**		
LDH(U/L)		51.17 ± 0.60			86.17 ± 2.50^**^				62.33 ± 1.09		136.33 ± 7.81^**^		
mALB (μg/ml)		290.33 ± 1.59			309.18 ± 1.41^**^				292.93 ± 1.85		313.59 ± 1.20^**^		
**B. 3-D printer emission exposure.**													
**3-D printer emission 1st exposure**														
	**2-h exposure**							**24-h post-exposure**						
	**Control**			**Exposure**				**Control**				**Exposure**		
Viability (%)	93.50 ± 1.00			86.25 ± 1.75				93.75 ± 0.25				94.75 ± 0.25		
LDH(U/L)	25.25 ± 10.09			9.25 ± 3.33				61.50 ± 5.50				56.5 ± 6.50		
mALB (μg/ml)	0.74 ± 0.10			0.82 ± 0.2				116.9 ± 1.32				115.38 ± 1.14		
uTP (mg/dl)	7.65 ± 0.22			7.67 ± 0.07				263.15 ± 13.96				254.20 ± 0.88		
**3-D printer emission 2nd exposure**														
	**2-h exposure**						**24-h post-exposure**							

	**Control**	**Exposure 1**	**Exposure 2**	**Control**	**Exposure 1**	**Exposure 2**
Viability (%)	86.25 ± 1.75	90.00 ± 1.00	85.00 ± 0.50	96.50 ± 0.50	93.00 ± 3.00_	92.25 ± 0.75
LDH(U/L)	56.00 ± 15.59	42.75 ± 5.31	40.75 ± 6.34	100.00 ± 30.00	71.50 ± 3.50	73.00 ± 5.00
mALB (μg/mL)	0.61 ± 0.05	0.400 ± 0.05 *	0.81 ± 0.14	118.02 ± 0.72	116.14 ± 2.13	117.02 ± 1.45
uTP (mg/dl)	7.90 ± 0.24	7.78 ± 0.14	8.03 ± 0.10	269.03 ± 0.63	271.75 ± 5.42	265.33 ± 0.50^*^

Data expressed as mean ± S.E; **p < 0.01; comparing with control; *p < 0.05; comparing with control. LDH (lactate dehydrogenase); mALB, microalbumin, uTP, total protein. A. Positive control AgNP, LDH (*n* = 6), uTP (*n* = 6); B. 3-D printer emission exposure. 2-h exposure, Viability (*n* = 2); LDH (*n* = 4), mALB (*n* = 4), uTP (*n* = 4). 24-h post-exposure. Viability (*n* = 2); LDH (*n* = 2), mALB (*n* = 2), uTP (*n* = 2).

### Cellular Mass and Particle Dose Estimation

The average mass concentrations in the air during the two 2-h exposure were 0.884 mg/m^3^; 100% deposition dose (mg/transwell) = 884 μg/m^3^ × 10 ml/min × 120 min = 1.06 μg. The deposit dose = 28% × 1.06 μg = 0.3 μg/well 28% = system deposition rate for nanomaterials with similar size (30 ± 5% for 30 nm silver nanoparticles in HIVIS system). For an average of 3 × 10^5^ cells per transwell, 318 ng/300,000 cells = 1.06 pg/cell. For a cellular dose, average 884 μg/m^3^ would be 55,048,022 particles for ABS. If you use C_m_= C_N_ × ρπ/6 × (d_m_)^3^ (Hinds, 1999, formula 4.53), where C_m_ = mass concentration (884 μg/m^3^)

ρ = density (ABS, 1.052/cc), d_m_ = diameter of average mass (30.8 nm), C_N_ = number concentration (55.048 × 10^6^/cc) 55,048,022 particles/300,000 cells = 183.5 particles/cell. The deposit particle dose = 28% × 183.5 particles/cell = 51.38 particles/cell.

### Inflammatory Cytokine Levels After AgNP and ABS Exposure

**FIGURE 4:**
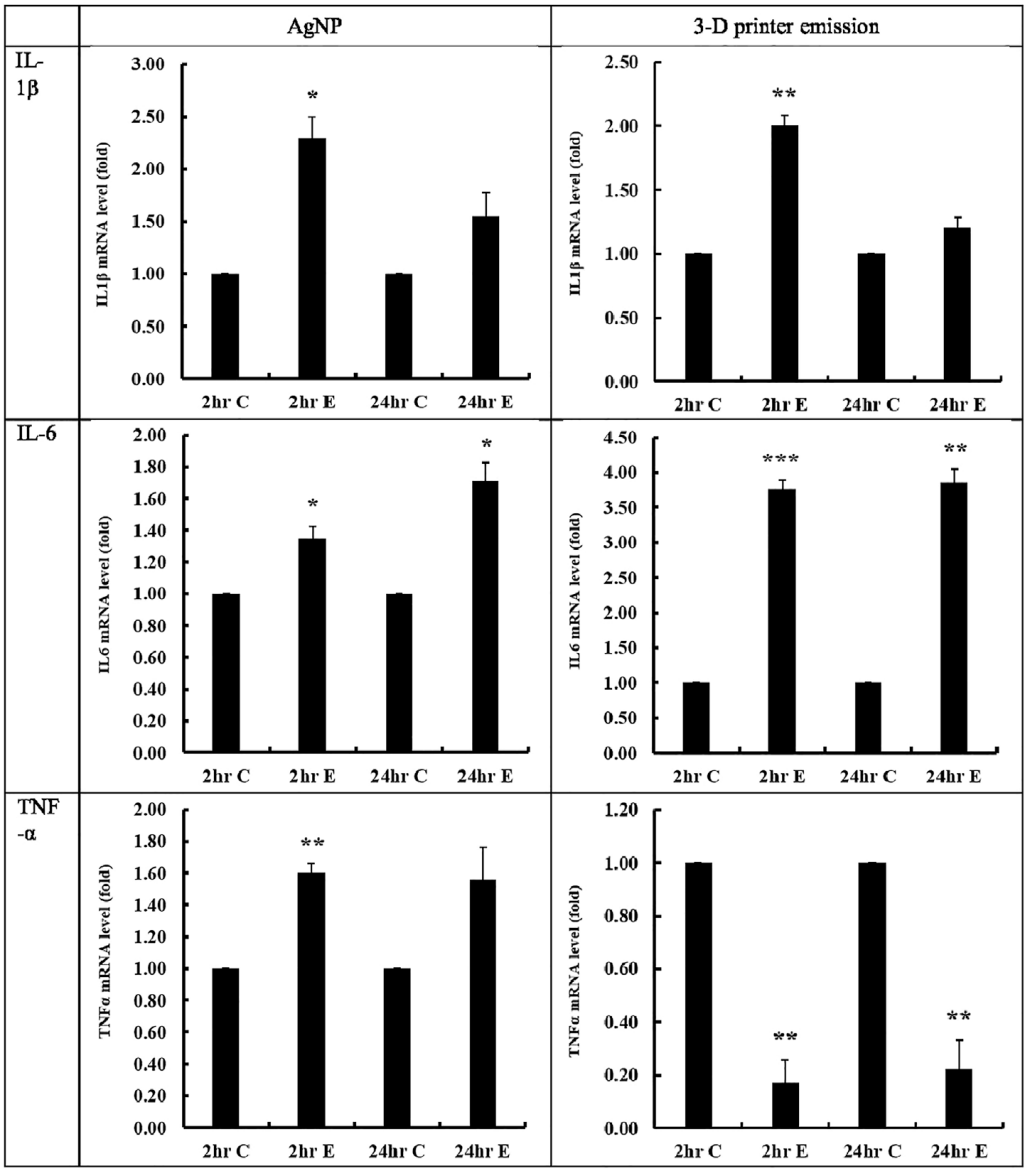
Inflammatory cytokine levels after AgNP and 3-D printer emission. mRNA levels of IL-1β, IL-6, and TNF-α were measured after 2-h exposure and post-exposure 24-h incubation. 2C, 2-h control; 2E, 2-h exposure; 24C, post-exposure 24-h control; 24E, post-exposure 24 h; *, *p* < 0.05; **, *p* < 0.01; ***. *p* < 0.001. *n* = 3).

After 2-h exposure of A549 cells to the positive control AgNP, the mRNA levels for IL-1b, IL-6, and TNF-α increased significantly (*p* < 0.05) and continued to do so 24-h post-exposure incubation (*p* < 0.05, except for TNF-α) (Figure 4). The mRNA levels of IL-1b and IL-6 increased significantly (*p* < 0.01–0.001) after 2-h exposure of A549 cells to ABS particles, as well as 24-h post-exposure incubation. In contrast to the AgNP aerosol challenge, the mRNA levels of TNF-α decreased significantly after 2 h of ABS particle exposure and even more so after 24-h of post-exposure incubation (Figure 4). The 2-h exposure of A549 cells to AgNP mediated inflammation-promoting signaling by increasing the initial response of IL-1β, IL-6, and TNF-α, and IL-6 could be involved continuously in the inflammatory response. In addition, exposure to 3-D printers using ABS filaments for 2 h increased the initial IL-1β and IL-6 level, which is involved in inflammation-related signaling and is thought to be involved in the inflammatory response through continuous secretion of IL-6. The reduction of TNF-α mRNA expression following ABS exposure may indicate the involvement of different inflammatory pathways from AgNP.

## Discussion

This study applied the HIVIS ALI exposure system to assess the cytotoxicity of 3-D printer emissions that used an ABS filament. The current on-site ALI system setup mimics actual human lung exposure to 3-D emissions. 1) The A549 cells are adenocarcinoma human alveolar epithelial cells that represent models of type II pulmonary epithelium. 2) In this study, 3-D emissions were delivered to A549 cells as-is, containing both aerosols with gaseous co-pollutants, which is similar to the delivery of such aerosols to human lungs. This is in contrast to the conventional approach of collecting 3-D emitted particles on a filter media, followed by multiple post-processing steps, such as extraction, resuspension, dispersion, and dosing of cells, that can introduce various artifacts and has little resemblance to the particle deposition in the airways. 3) The primary deposition mechanism to A549 cells in HIVIS ALI is diffusion (as explained in Supplement information Supplementary Table S1), which is also a major deposition mechanism of nanoparticles in the alveolar region. The particle deposition pattern in our ALI system, estimated at 28%, is similar to the modeled deposition of 3-D printer aerosols in the human lungs from the MPPD (multiple pathway particle dosimetry) model 3.04 (Anjilvel and Asgharian, 1995), which (the model) predicted 26% deposition of ABS particles (with CMD 30 nm) in the alveolar region.

Our particle size measurement of 3-D printing emissions from the inside of the 3-D printer-1 enclosure indicated that 3-D printer-1 emissions were generated as vapors of semi-volatile organics because they could not be initially measured with DMAS. However, as vapors cooled off, they condensed to form the nucleation stage, a size that is measurable by DMAS and further reached the coagulation stage where their size is measurable by OPC or a dust monitor. In contrast, emissions taken from 3-D printer-2 showed 1.73–2.50 × 106 particles/cc depending on the 3-D printing process. Our UFP concentration of 16,000 particles/cc during printer operations was similar to the range reported by other studies (Stephens et al., 2013; Afshar-Mohajer et al., 2015; Deng et al., 2016; Steinle, 2016; Mendes et al., 2017; Vance et al., 2017). Total VOC (TVOC) concentrations from 3-D printing emission reported in another paper (Kim et al., 2021 submitted) were also similar to concentrations of 102–103 μg/m^3^ reported previously (Azimi et al., 2016; Steinle, 2016; Floyd et al., 2017; Mendes et al., 2017).

UFP particles emitted from 3-D printers have been subjected to toxicological studies *in vivo* and *in vitro*. Three types of 3-D printer-emitted particles were collected on polytetrafluoroethylene (PTFE) membrane filters and extracted to assess their cytotoxicity in different cell types: at alveolar macrophages (NR8383, CRL 2192) and A549 cells. All three types (ABSc, ABSd, and PLA) of particles statistically significantly decreased cell viability for A549 and NR8383 cells compared to the controls at the indicated concentrations of 20 mg/ml for ABSd, 59 mg/ml for ABSc, and 2.3 mg/ml for PLA, with no significant differences between cell lines (Zhang et al., 2019). Also, these same authors exposed the particles to the mice’s respiratory tract *via* intratracheal instillation and obtained bronchoalveolar lavage fluid at 24-h post-exposure. The three types of particles showed an inflammatory response with increased neutrophil numbers (Zhang et al., 2019).

In another study, ABS and polycarbonate 3-D printer-emitted particles were collected into the culture medium from a 3-D printer located inside a chamber. The collected particles were tested for their toxicity in human small airway epithelial cells. After 24-h post-exposure, both PC and ABS emissions induced significant dose-dependent cytotoxicity from 5 × 106 to 2 × 107 particles/cm^2^ for PC and 1–5 × 10^6^ particles/cm^2^ for ABS, oxidative stress, apoptosis, necrosis, and production of pro-inflammatory cytokines and chemokines in human small airway epithelial cells. They found a significant (*p* < 0.0001) positive correlation between the IL-12p70, IL-13, IL-16, IL-1β, IL-1α, IL-6, IL-8, and TNF-α levels (Farcas et al., 2019). ABS emissions generated from a desktop 3-D printer were used for rat inhalation exposure. Three-hour exposure of ABS particles to Sprague–Dawley rats increased a mean arterial pressure (125 ± 4 mm Hg) when compared with the control (94 ± 3 mm Hg). It resulted in an impairment of endothelium-dependent arteriolar dilation after 24 h, suggesting alterations in peripheral microvascular resistance and reactivity following ABS emissions (Stefaniak et al., 2017). In our study, ABS exposure did not show any significant cytotoxicity to A549 cells, yet exposure to ABS emissions increased initial IL-1β and IL-6 mRNA levels, which are involved in inflammation-related signaling and are thought to be involved in the inflammatory response through continuous secretion of IL-6. Reduction of the TNF-α mRNA level following ABS exposure may indicate involvement of different mechanistic pathways, which is interesting to study.

Although the cytotoxicity of 3-D printer emission has not been tested by the ALI system, carbonaceous nanomaterials and other nanomaterials have been tested by the ALI system. To optimize an ALI system for flow-through inhalation exposure, Leibrock et al. (2020) exposed CeO_2_ nanoparticle aerosols to A549 cells using the ALI system. Five influential instrumental and physiological parameters, including exposure duration, relative humidity, temperature, CO_2_ concentration, and flow rate, were evaluated. CeO_2_ aerosol exposure to A549 cells reached values similar to those found in a recent subacute rat inhalation study *in vivo*. Furthermore, their approach provided useful guidance on standardizing and validating the use of an ALI system (Leibrock et al., 2020). We also studied the influence of flow rate and exposure duration, but on-site tests limited further appropriate parametric studies for relative humidity and CO_2_ concentration. Acute toxicity of MWCNTs was evaluated in another study using an air–liquid interface at concentrations of 0.16 and 0.34 µg/cm^2^ after 24 h along with two positive controls Dörentruper Quartz (DQ12) and asbestos. MWCNTs caused neither increased release of lactate dehydrogenase (LDH) nor alterations in inflammatory responses, as measured by RNA expression and protein secretion of the cytokines IL-6, IL-8, CXCL10, IL-1β, and TGF-β and oxidative stress markers HMOX-1 and SOD-2 (Beyeler et al., 2018). Although the delivered doses for MWCNTs were higher than in our study (0.3 µg/well 4.7 cm^2^), no cytotoxicity was observed in both studies, but 3-D emission induced IL-1, IL-6 (*p* < 0.05), and TNF-α mRNAs. In another study, MWCNTs at concentration of 2–10 µg/cm^2^ were tested in co-cultured cells, alveolar epithelial cells (A549), fibroblasts (MRC-5), and macrophages (differentiated THP-1) using the VITROCELL^®^ Cloud system after acute (24 h, one exposure) and prolonged (96 h, repeated exposures) exposures. The acute or prolonged exposure to different concentrations of the tested MWCNTs did not induce cytotoxicity or apparent profibrotic response; however, it suggested the onset of pro-inflammatory response (Barosova et al., 2020). Future studies should investigate the influence of important variables of exposure duration and incubation duration post-exposure on cytotoxicity and other relevant biomarkers (such as induction of pro-inflammatory responses) with different ALI systems. Previous investigations have shown that several types of nanoparticles did not interfere with the LDH assay (Guadagnini et al., 2015; Andraos et al., 2020; Peterson et al., 2020). Our study (Andraos et al., 2020), and the one by Guadagnini et al. (2015) has shown that the LDH assay is reasonably robust from interference from several nanoparticles, except MWCNTs (Wang et al., 2012); therefore, it could also be used to assess the cytotoxicity of the 3-D printer emissions. The use of the basal side medium in the cytotoxicity test could further reduce the interference from the presence of nanoparticles. Despite several control experiments adopted in this experiment, there would be more potential artifacts (assay-specific and nanoparticle-specific) in the test system. The thoughtful design of control experiments, including flow rate, humidity, temperature, and assay-specific controls, should be considered.

In this 3-D printer emission *in vitro* toxicity study, the HIVIS ALI exposure system was used to evaluate the cytotoxicity of 3-D printer particle emissions. 3-D printer particle emission contained not only particulate materials (mainly UFPs) but also gaseous chemicals such as PAH, formaldehyde, and VOCs. These UPFs and chemicals are expected to deposit to the respiratory tract, primarily *via* diffusion. The main deposition mechanism in the HIVIS ALI system we used for cytotoxicity evaluation was also diffusion; thus, it mimics somewhat the nanoparticle delivery to the lower respiratory tract of human lungs. Our system deposition efficiency using 20–30 nm silver nanoparticles ranged 30 ± 5%; thus, when assuming that particle density is not critical in the diffusion regime, the deposition of 3-D particle emission (∼30 nm) would be similar to the particle having a similar diameter as silver nanoparticles (20–30 nm). Although we estimated the deposited dose as 1.06 pg/cell, it would be possible to measure the deposited dose to cells once a mass spectrometry method is developed to quantify ABS signature exposure markers in cells. As dose calculations reveal, the cellular dose in the current ALI system was much lower than in previous 3-D printer emission studies that relied on collected particles on filters.

The concentration and duration of exposure applied in this study may not be sufficient to induce cytotoxicity. Although the deposition rate (∼30%) in the HIVIS system is not low, the deposited mass dose may not be sufficient to elicit a cytotoxic response. This may actually be a more realistic workers’ exposure representation of what happens in the human airways when workers get exposed to such levels—a mild pro-inflammatory response that does not do immediate harm but that seems still unhealthy if repeated over many years of work. By printing more compact objects than current irregularly shaped objects, we could see consistently higher emissions. In this case, we may see the induction of cytotoxicity. The general cytotoxicity biomarkers we used were trypan blue exclusion, LDH release, microprotein, and total protein for cytotoxicity evaluation. Despite the fact that the absence of cytotoxicity increased the levels of IL-1b and IL-6 cytokine mRNA, our hyperspectral microscopy confirms that ABS UFPs were associated with A549 cells and could be internalized to cytoplasm and nucleus regions. Post-exposure of 24 h showed the internalization of many more UFPs into the cytoplasm as well as the nuclear region. Future studies should include internalization and intercellular location of 3-D printer-emitted particles, higher deposited doses, and measuring cytokine production (measuring the proteins) in combination with genotoxicity. Most *in vitro* assays rely on immortalized cell lines, which tend to be more resilient and less sensitive to injury. The use of more sensitive human-derived primary cell lines and co-cultures with an ALI system may further increase their relevance. After recognizing these sarcoma incidences and receiving our recommendation on the emissions measurements and recommendations, the workplace implemented mitigation of emissions from 3-D printers by isolating 3-D printers inside an enclosed space with dedicated local exhaust ventilation. Thus, we will conduct further studies after getting permission from the workplace to assess exposures and hazard levels.

In a concomitant study, along with this study, we conducted a comprehensive characterization of exposures, including VOCs, particle number and mass concentration, size distribution, and elemental composition/morphology (Kim et al., 2022). Exposure assessment documented low levels of total suspended particulates (9–12.5 µg/m^3^), minimal levels (1.93–4 ppm) of TVOC as measured by a real-time monitor, and formaldehyde (2.5–21.7 ppb), with no detectable levels of benzene and styrene GC-FID. PTR-TOF-MS analysis of grab samples detected various chemicals at low concentrations, the majority of which were in the low ppb. UFPs emitted from 3-D printers had an average particle size of 30 nm CMD and 71 nm MMAD, and their size continued to increase after the termination of 3-D printing through the night until morning of the next day.

Furthermore, the HIVIS ALI test system was employed at an actual workplace that used 3-D printers; therefore, exposures delivered to ALI are somewhat practical in exposure profile, exposure scenarios, and delivered dose. Two consecutive cytotoxicity evaluation tests using an on-site ALI system at airborne concentrations of 810 and 957 mg/m^3^ for 2-h exposure to 3-D printer-emitted particles, and 24-h post-exposure incubation, resulted in no significant cytotoxicity, as measured by common cellular toxicity evaluation endpoints of cell viability/energetics, and cell necrosis, as well as microprotein expression. Despite the lack of measurable cytotoxicity, ABS particles were observed to interact with and be internalized by A549 cells after 2-h exposure and thereafter 24-h incubation period, evidenced by the hyperspectral microscopy, and to induce expression of important pro-inflammatory cytokine mRNAs.

It is important to note that this ALI system probes acute effects on cells following a single 2-hr short-term exposure. Future studies should explore chronic effects on cells following, to the extent possible, multiple exposures of longer duration.

## Data Availability

The original contributions presented in the study are included in the article/Supplementary Material, further inquiries can be directed to the corresponding author.
