# Supplementary material for: On-Site Deployment of an Air-Liquid-Interphase Device to Assess Health Hazard Potency of Airborne Workplace Contaminants: The Case of 3-D Printers
Source: Front Toxicol. 2022 Mar 25;4:818942. doi: 10.3389/ftox.2022.818942 (PMC8990836; doi:10.3389/ftox.2022.818942)

Supplement Table S1. Impaction deposition calculation. 20^o^C air, the distance from circular nozzle to impaction plate was 1.5-2.0 times of diameter of nozzle. Reynolds number were 500-3000, maximum 4000. The impaction was calculated based formula shown below (5.28) and (5.29) in Aerosol Technology (Hinds, 1999). The results indicated that 30 nm particle required 147 ml/min (light blue color shade) to achieve 50% deposition by impaction. In our condition of flow rate 10 ml/min, 50% of deposition by impaction required particle size 104 ㎛ (green color shade).


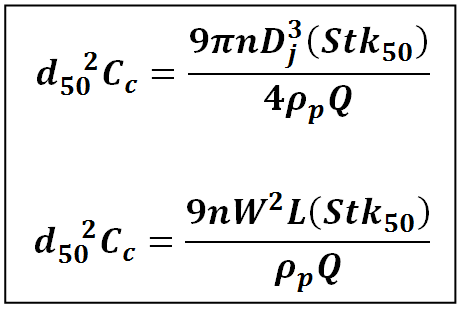


| Density of particle (ρ_p_) (kg/m^3^) | Kinematic Viscosity of Air (ρ) (m^2^/S) | circular nozzle diameter (Dj) (m) | Minimum nozzle gap (m) | No. of nozzle | Flow rate (Q) (LPM) | Flow rate (m^3^/s) | Flow velocity (V) (m/s) | Reynolds number | Stokes No. for 50% collection efficiency (Stk_50_) | coefficient of viscosity (η;20℃, air) (Nsec/㎥) | d_50_^2^C_c (m)_ | d_50_^2^C_c (㎛)_ | particle diameter having 50% collection efficiency (d_50_) _(㎛)_ |
| --- | --- | --- | --- | --- | --- | --- | --- | --- | --- | --- | --- | --- | --- |
| 1070 | 0.00001511 | 0.004 | 0.006 | 1 | 0.005 | 8.33E-08 | 0.0066 | 1.8 | 0.24 | 0.0000181 | 2.20E-08 | 2.20E-02 | **148.379** |
| 1070 | 0.00001511 | 0.004 | 0.006 | 1 | 0.01 | 1.67E-07 | 0.0133 | 3.5 | 0.24 | 0.0000181 | 1.10E-08 | 1.10E-02 | **104.897** |
| 1070 | 0.00001511 | 0.004 | 0.006 | 1 | 0.03 | 5.00E-07 | 0.0398 | 10.5 | 0.24 | 0.0000181 | 3.67E-09 | 3.67E-03 | **60.529** |
| 1070 | 0.00001511 | 0.0001 | 0.00015 | 1 | 0.147 | 2.45E-06 | 311.9437 | 2064.5 | 0.24 | 0.0000181 | 1.17E-14 | 1.17E-08 | **0.030** |
| 1070 | 0.00001511 | 0.002 | 0.003 | 1 | 0.005 | 8.33E-08 | 0.0265 | 3.5 | 0.24 | 0.0000181 | 2.75E-09 | 2.75E-03 | **52.409** |
| 1070 | 0.00001511 | 0.002 | 0.003 | 1 | 0.01 | 1.67E-07 | 0.0531 | 7.0 | 0.24 | 0.0000181 | 1.38E-09 | 1.38E-03 | **37.036** |
| 1070 | 0.00001511 | 0.002 | 0.003 | 1 | 0.03 | 5.00E-07 | 0.1592 | 21.1 | 0.24 | 0.0000181 | 4.59E-10 | 4.59E-04 | **21.350** |

Supplement Table S2. Toxicity test depending on flow rates. The cells were exposed to the 3-D printer emissions for 2-hr at an airflow rate of 5 and 10 ml/min at room temperature. The cell-free medium and the incubator control cells were placed in an incubator, and the air control was placed outside the incubator. After 2-hr exposure, Cytotoxicity endpoints were evaluated by the lactate dehydrogenase (LDH) assay, total, and microprotein (uTP).

| Cell free medium | LDH (U/L) | uTP (mg/dL) |
| --- | --- | --- |
| 1 | 38 | 284.35 |
| 2 | 41 | 279.58 |
| mean ± S.E | 39.5 ± 1.5 | 281.97 ±2.39 |
| Incubator control |  |  |
| 1 | 61 | 282.61 |
| 2 | 52 | 279.58 |
| 3 | 54 | 291.21 |
| 4 | 58 | 299.69 |
| 5 | 61 | 295.17 |
| 6 | 56 | 305.20 |
| mean ± S.E | 57.00 ± 1.51 | 292.24 ± 4.03 |
| Air control |  |  |
| 1 | 58 | 282.92 |
| 2 | 55 | 283.91 |
| 3 | 64 | 317.57 |
| 4 | 54 | 275.12 |
| 5 | 55 | 292.76 |
| 6 | 74 | 329.64 |
| mean ± S.E | 60.00 ± 3.17 | 296.99 ± 8.86 |
| 5cc/min |  |  |
| 1 | 56 | 283.60 |
| 2 | 50 | 281.00 |
| 3 | 56 | 292.45 |
| 4 | 55 | 286.32 |
| 5 | 55 | 286.32 |
| 6 | 57 | 290.53 |
| mean ± S.E | 54.83 ± 1.01 | 286.70 ± 1.73 |
| 10 cc/min |  |  |
| 1 | 54 | 283.91 |
| 2 | 50 | 276.11 |
| 3 | 48 | 280.44 |
| 4 | 59 | 295.54 |
| 5 | 56 | 291.89 |
| 6 | 50 | 295.11 |
| mean ± S.E | 52.83 ± 1.72 | 287.17 ± 3.33 |

(mean ± S.E)

Supplement Figure S1. 3-D printed objects.

| A. Printer 1 | B. Printer 2 |
| --- | --- |
| 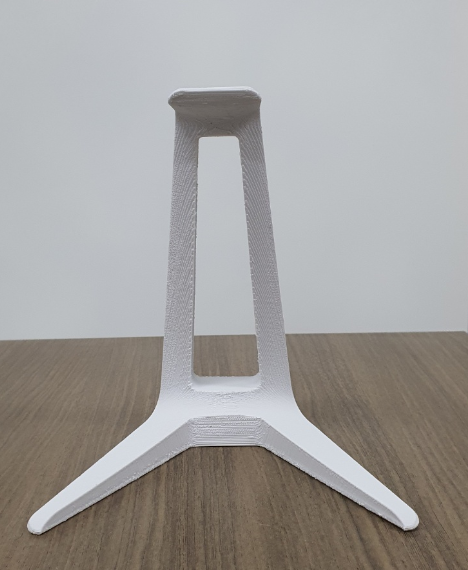 | 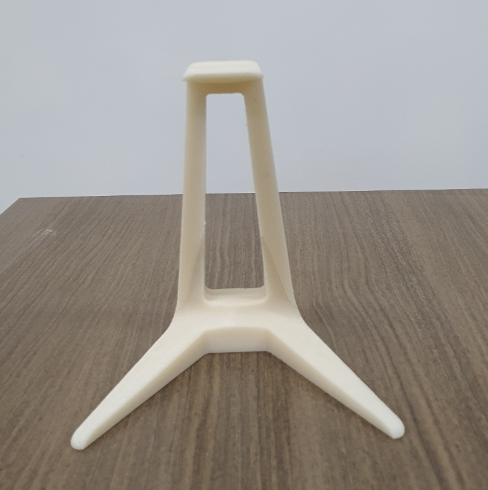 |

**Supplement Figure 2.** Workplace layout, 3-D printer location, and sampling locations. Dimension: 6.75 m x11.25 m x2.72 m. DMAS, Differential mobility analyze system; PAS, photoelectric aerosol sensor; PM sensor, particulate matter sensor.


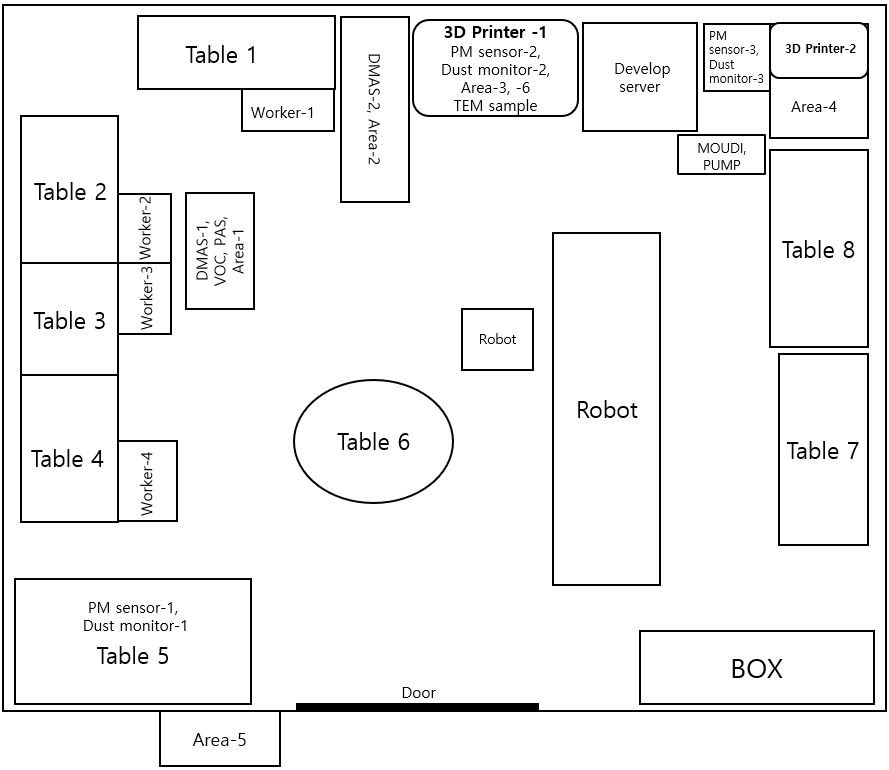

Supplement: Supplementary file 1 [file DataSheet1.docx]
